# Supplementary material for: HIVprotI: an integrated web based platform for prediction and design of HIV proteins inhibitors
Source: J Cheminform. 2018 Mar 9;10:12. doi: 10.1186/s13321-018-0266-y (PMC5845081; doi:10.1186/s13321-018-0266-y)
Supplement: Supplementary file 2 — Additional file 2. Source code of HIVProtI web server. [file 13321_2018_266_MOESM2_ESM.zip › HIVprotI_Source-code/hivprotI/weka-3-6-6/doc/weka/core/CheckScheme.html]

CheckScheme


---


|  |  |  |
| --- | --- | --- |
| **Overview** \| **Package** \| **Class** \| **Tree** \| **Deprecated** \| **Index** \| **Help** \| **Weka's home** | |  |
| **PREV CLASS**   **NEXT CLASS** | **FRAMES**    **NO FRAMES**     **All Classes** |  |
| SUMMARY: NESTED \| FIELD \| CONSTR \| METHOD | DETAIL: FIELD \| CONSTR \| METHOD |  |


---


## weka.core Class CheckScheme

```
java.lang.Object
  weka.core.Check
      weka.core.CheckScheme
```

**All Implemented Interfaces:**: OptionHandler, RevisionHandler

**Direct Known Subclasses:**: CheckAssociator, CheckAttributeSelection, CheckClassifier, CheckClusterer, CheckKernel

---

``` public abstract class CheckScheme extends Check ```

Abstract general class for testing schemes in Weka. Derived classes are
also used for JUnit tests.

**Version:**
:   $Revision: 1.4 $

**Author:**
:   FracPete (fracpete at waikato dot ac dot nz)

**See Also:**: `TestInstances`

---

| **Nested Class Summary** | |
| --- | --- |
| `static class` | `CheckScheme.PostProcessor`             a class for postprocessing the test-data |


**Constructor Summary**

`CheckScheme()`


| **Method Summary** | |
| --- | --- |
| `static java.lang.String` | `attributeTypeToString(int type)`             returns a string representation of the attribute type |
| `abstract  void` | `doTests()`             Begin the tests, reporting results to System.out |
| `int` | `getNumDate()`             returns the current number of date attributes |
| `int` | `getNumInstances()`             Gets the current number of instances to use for the datasets. |
| `int` | `getNumInstancesRelational()`             returns the current number of instances in relational/bag attributes to produce |
| `int` | `getNumNominal()`             returns the current number of nominal attributes |
| `int` | `getNumNumeric()`             returns the current number of numeric attributes |
| `int` | `getNumRelational()`             returns the current number of relational attributes |
| `int` | `getNumString()`             returns the current number of string attributes |
| `java.lang.String[]` | `getOptions()`             Gets the current settings of the CheckClassifier. |
| `CheckScheme.PostProcessor` | `getPostProcessor()`             returns the current PostProcessor, can be null |
| `java.lang.String` | `getWords()`             returns the words used for assembling strings in a comma-separated list. |
| `java.lang.String` | `getWordSeparators()`             returns the word separators (chars) to use for assembling strings. |
| `boolean` | `hasClasspathProblems()`             returns TRUE if the classifier returned a "not in classpath" Exception |
| `java.util.Enumeration` | `listOptions()`             Returns an enumeration describing the available options. |
| `void` | `setNumDate(int value)`             sets the number of data attributes |
| `void` | `setNumInstances(int value)`             Sets the number of instances to use in the datasets (some classifiers might require more instances). |
| `void` | `setNumInstancesRelational(int value)`             sets the number of instances in relational/bag attributes to produce |
| `void` | `setNumNominal(int value)`             sets the number of nominal attributes |
| `void` | `setNumNumeric(int value)`             sets the number of numeric attributes |
| `void` | `setNumRelational(int value)`             sets the number of relational attributes |
| `void` | `setNumString(int value)`             sets the number of string attributes |
| `void` | `setOptions(java.lang.String[] options)`             Parses a given list of options. |
| `void` | `setPostProcessor(CheckScheme.PostProcessor value)`             sets the PostProcessor to use |
| `void` | `setWords(java.lang.String value)`             Sets the comma-separated list of words to use for generating strings. |
| `void` | `setWordSeparators(java.lang.String value)`             sets the word separators (chars) to use for assembling strings. |

**Methods inherited from class weka.core.Check**

`getDebug, getSilent, setDebug, setSilent`

**Methods inherited from class java.lang.Object**

`equals, getClass, hashCode, notify, notifyAll, toString, wait, wait, wait`

**Methods inherited from interface weka.core.RevisionHandler**

`getRevision`

**Constructor Detail**

### CheckScheme

```
public CheckScheme()
```


**Method Detail**

### listOptions

```
public java.util.Enumeration listOptions()
```

:   Returns an enumeration describing the available options.

    :   **Specified by:**: `listOptions` in interface `OptionHandler` **Overrides:**: `listOptions` in class `Check`
    :   **Returns:**: an enumeration of all the available options.

---


### setOptions

```
public void setOptions(java.lang.String[] options)
                throws java.lang.Exception
```

:   Parses a given list of options.

    :   **Specified by:**: `setOptions` in interface `OptionHandler` **Overrides:**: `setOptions` in class `Check`
    :   **Parameters:**: `options` - the list of options as an array of strings **Throws:**: `java.lang.Exception` - if an option is not supported

---


### getOptions

```
public java.lang.String[] getOptions()
```

:   Gets the current settings of the CheckClassifier.

    :   **Specified by:**: `getOptions` in interface `OptionHandler` **Overrides:**: `getOptions` in class `Check`
    :   **Returns:**: an array of strings suitable for passing to setOptions

---


### setPostProcessor

```
public void setPostProcessor(CheckScheme.PostProcessor value)
```

:   sets the PostProcessor to use

    :   **Parameters:**: `value` - the new PostProcessor **See Also:**: `m_PostProcessor`

---


### getPostProcessor

```
public CheckScheme.PostProcessor getPostProcessor()
```

:   returns the current PostProcessor, can be null

    :   **Returns:**: the current PostProcessor

---


### hasClasspathProblems

```
public boolean hasClasspathProblems()
```

:   returns TRUE if the classifier returned a "not in classpath" Exception

    :   **Returns:**: true if CLASSPATH problems occurred

---


### doTests

```
public abstract void doTests()
```

:   Begin the tests, reporting results to System.out

    :   **Specified by:**: `doTests` in class `Check`

---


### setNumInstances

```
public void setNumInstances(int value)
```

:   Sets the number of instances to use in the datasets (some classifiers
    might require more instances).

    :   **Parameters:**: `value` - the number of instances to use

---


### getNumInstances

```
public int getNumInstances()
```

:   Gets the current number of instances to use for the datasets.

    :   **Returns:**: the number of instances

---


### setNumNominal

```
public void setNumNominal(int value)
```

:   sets the number of nominal attributes

    :   **Parameters:**: `value` - the number of nominal attributes

---


### getNumNominal

```
public int getNumNominal()
```

:   returns the current number of nominal attributes

    :   **Returns:**: the number of nominal attributes

---


### setNumNumeric

```
public void setNumNumeric(int value)
```

:   sets the number of numeric attributes

    :   **Parameters:**: `value` - the number of numeric attributes

---


### getNumNumeric

```
public int getNumNumeric()
```

:   returns the current number of numeric attributes

    :   **Returns:**: the number of numeric attributes

---


### setNumString

```
public void setNumString(int value)
```

:   sets the number of string attributes

    :   **Parameters:**: `value` - the number of string attributes

---


### getNumString

```
public int getNumString()
```

:   returns the current number of string attributes

    :   **Returns:**: the number of string attributes

---


### setNumDate

```
public void setNumDate(int value)
```

:   sets the number of data attributes

    :   **Parameters:**: `value` - the number of date attributes

---


### getNumDate

```
public int getNumDate()
```

:   returns the current number of date attributes

    :   **Returns:**: the number of date attributes

---


### setNumRelational

```
public void setNumRelational(int value)
```

:   sets the number of relational attributes

    :   **Parameters:**: `value` - the number of relational attributes

---


### getNumRelational

```
public int getNumRelational()
```

:   returns the current number of relational attributes

    :   **Returns:**: the number of relational attributes

---


### setNumInstancesRelational

```
public void setNumInstancesRelational(int value)
```

:   sets the number of instances in relational/bag attributes to produce

    :   **Parameters:**: `value` - the number of instances

---


### getNumInstancesRelational

```
public int getNumInstancesRelational()
```

:   returns the current number of instances in relational/bag attributes to produce

    :   **Returns:**: the number of instances

---


### attributeTypeToString

```
public static java.lang.String attributeTypeToString(int type)
```

:   returns a string representation of the attribute type

    :   **Parameters:**: `type` - the attribute type to get a string rerpresentation for **Returns:**: the string representation

---


### setWords

```
public void setWords(java.lang.String value)
```

:   Sets the comma-separated list of words to use for generating strings. The
    list must contain at least 2 words, otherwise an exception will be thrown.

    :   **Parameters:**: `value` - the list of words **Throws:**: `java.lang.IllegalArgumentException` - if not at least 2 words are provided

---


### getWords

```
public java.lang.String getWords()
```

:   returns the words used for assembling strings in a comma-separated list.

    :   **Returns:**: the words as comma-separated list

---


### setWordSeparators

```
public void setWordSeparators(java.lang.String value)
```

:   sets the word separators (chars) to use for assembling strings.

    :   **Parameters:**: `value` - the characters to use as separators

---


### getWordSeparators

```
public java.lang.String getWordSeparators()
```

:   returns the word separators (chars) to use for assembling strings.

    :   **Returns:**: the current separators


---


|  |  |  |
| --- | --- | --- |
| **Overview** \| **Package** \| **Class** \| **Tree** \| **Deprecated** \| **Index** \| **Help** \| **Weka's home** | |  |
| **PREV CLASS**   **NEXT CLASS** | **FRAMES**    **NO FRAMES**     **All Classes** |  |
| SUMMARY: NESTED \| FIELD \| CONSTR \| METHOD | DETAIL: FIELD \| CONSTR \| METHOD |  |


---
